# Editorial Comment on “Recurrent multiple liver metastases of clear cell renal cell carcinoma with a significant response to sunitinib after nivolumab treatment: A case report”

**DOI:** 10.1002/iju5.12553

**Published:** 2022-11-08

**Authors:** Koji Hatano

**Affiliations:** ^1^ Department of Urology Osaka University Graduate School of Medicine Suita Japan

The advent of tyrosine kinase inhibitors (TKIs) and immune checkpoint inhibitors (ICIs) has increased treatment options for metastatic renal cell carcinoma (mRCC). In this case, a patient with mRCC who received sunitinib as 5th line therapy after 4th line nivolumab showed significant tumor shrinkage, and maintained partial response for 34 months.^1^ Interestingly, other TKIs, such as axitinib and cabozantinib, given as 2nd and 3rd line therapy, showed only modest efficacy. The authors mentioned that the efficacy of sunitinib may have occurred as a result of the alteration of the tumor's immunological microenvironment by nivolumab.^1^ To date, several studies have reported the efficacy of TKIs in mRCC following ICIs, with objective response rates ranging from 27% to 50%.^1^

Currently, the International Metastatic Renal Cell Carcinoma Database Consortium (IMDC) prognostic criteria are used to stratify patients into three risk groups, based on clinical (performance status, interval from diagnosis to treatment), and laboratory (hypercalcemia, anemia, thrombocytopenia, neutrophilia) variables. Neutrophilia is associated with an inflammatory response, which may lead to the suppression of lymphocytes, natural killer cells, and activated T cells. In some solid tumors, including RCC, elevated systemic host inflammation markers such as CRP and neutrophil‐to‐lymphocyte ratio (NLR) have been shown to be associated with poor prognosis. The state of systemic inflammation can be monitored by changes in NLR in response to treatment. Templeton *et al*. reported that in mRCC patients treated with TKIs, an early decrease in NLR was associated with a favorable outcome compared to no change, while an increase in NLR was associated with a worse outcome.^2^ Lalani *et al*. reported that in mRCC patients treated with ICIs, an early decrease in NLR at 6 weeks was associated with a better prognosis.^3^ Nishiyama *et al*. also reported that in patients with mRCC treated with nivolumab, a NLR of ≥3 at 4 weeks after treatment initiation may be a predictor of poor prognosis.^4^ In this case, NLR temporarily increased with fever 4 weeks after sunitinib initiation, but it decreased after the fever resolved. This may be the result of systemic inflammatory changes.

## Conflict of interest

KH has received grants/research supports from Astellas Pharma Inc.
